# Status and situation of postgraduate medical students in China under the influence of COVID-19

**DOI:** 10.1136/postgradmedj-2020-137763

**Published:** 2020-05-13

**Authors:** Shunda Wang, Menghua Dai

**Affiliations:** Department of General Surgery, Peking Union Medical College Hospital,Chinese Academy of Medical Sciences and Peking Union Medical College, Beijing, China; Graduate School, Chinese Academy of Medical Sciences and Peking Union Medical College, Beijing, China; Department of General Surgery, Peking Union Medical College Hospital,Chinese Academy of Medical Sciences and Peking Union Medical College, Beijing, China

**Keywords:** epidemiology, medical education & training, preventive medicine

## Abstract

At the beginning of 2020, the outbreak of COVID-19 in China has brought great impact on the society, economy and life. This article introduces current status of Chinese postgraduate medical students under this epidemic situation in combination with the author’s own experience from four aspects: professional spirit, professional knowledge, learning status and protective measures.

## Introduction

A novel coronavirus has been discovered and confirmed since the first case of unidentified pneumonia was confirmed in Wuhan, China, in December 2019.^1  2^ The disease caused by this novel virus was officially named COVID-19 by the WHO on 12 January 2020. Since the outbreak in China, the numbers of confirmed cases and deaths have rapidly increased. COVID-19 has been clarified as a grade B infectious disease, others of which include severe acute respiratory syndrome and highly pathogenic avian influenza, and is treated according to the protocol for grade A infectious diseases. COVID-19 is the seventh known coronavirus-induced disease that involves infection of the respiratory system in human beings. The other two potentially life-threatening coronavirus-induced diseases are severe acute respiratory syndrome and Middle East respiratory syndrome.^3  4^ This novel coronavirus-induced pneumonia is transmitted from person to person and is highly infectious, with high susceptibility among the general population. The coronavirus responsible for COVID-19 has a long incubation period and diverse clinical features, seriously impacting normal work and life throughout the country. As of 13 April 2020, COVID-19 had been recognised in over 200 countries, with a total of 1 784 364 laboratory-confirmed cases and 111 832 deaths, and these numbers have since continued to rise.

On 23 January 2020, the Chinese government immediately blocked the city of Wuhan and cut off all outside contact to stop the spread of COVID-19. Other cities successively announced closure of public places and restricted the flow of people. At the time of this writing, the Chinese Ministry of Education had stated that no student was allowed to return to school until further notification. Some postgraduate medical students residing at school were isolated in safe places. Some others who had returned home for holiday were restricted to their local residence and prohibited to return to the hospital or medical school for studies or clinical work. We herein describe the status and situation of postgraduate medical students in China under the influence of COVID-19.

## Encouragement and promotion of the professional spirit of postgraduate medical students

At the frontline of the fight against COVID-19, many medical staff members around the country have devoted their full power without hesitation while ignoring their own personal safety. Their teachers, colleagues and friends have also participated in this battle. Such behaviour demonstrates the humanitarian nature of medicine, which involves healing the wounded and rescuing the dying. This vivid lesson helps medical students to internalise medical ethical principles through emotional penetration and thus deepens their understanding and strengthens their beliefs. It benefits society to cultivate a spirit of benevolence among medical students and to train postgraduate medical students to engage in positive behaviour. In recent years, the position of the medical humanities in medical education has gradually improved. The combination of medical humanities and medical knowledge is regarded as a successful medical education, which manifests scientific and human brilliance. Such education could help medical students to realise the transformation from medical ethical cognition to medical ethical behaviour in their future career.

## Use of professional knowledge to assist others

Medical students can help their relatives and friends to recognise the symptoms of pneumonia early according to their professional knowledge. The diagnosis of COVID-19 is based on a combination of epidemiological information, clinical symptoms, CT imaging findings and laboratory tests according to the standards of either the WHO or the National Health Commission of China. Although medical students were not in the hospital and had no access to CT or test kits, they generally have a higher level of professional judgement than people in the general population with respect to medical knowledge and patients’ symptoms. For example, if a person within a medical student’s neighbourhood develops a fever and cough and has a travel history from Wuhan, the student can advise him or her to go to the hospital in a timely manner. Postgraduate medical students can also educate the people around them, which helps the public to realise the importance of prevention and comply with regulations formulated by the country. Medical students can also serve as volunteers within the community and use their professional knowledge to make more contributions to community residents.

## Non-stop learning despite suspension of classes

The sudden outbreak of this novel coronavirus disrupted normal teaching and studying in the field of medical education. Non-stop learning via online teaching despite suspension of classes was put forward by the ministry of education. During the disease outbreak, online lectures and learning tutorials were adopted to avoid unnecessary aggregation of people and the associated risk of infection.^5^ Basic medical courses such as physiology, pathology and biology are relatively easy to study by video or electronic books. However, clinical medicine courses such as surgery are not suitable for online study. Because medicine is a practical science, it cannot break away from clinics and patients, and even simulation training cannot achieve a real-world effect. Many universities lack the ability to use the computers or software required to conduct online teaching courses, record teaching videos and prepare teaching documents such as text, picture, audio and animation. Students living in rural areas with underdeveloped networks and poor hardware facilities may find it difficult to meet the requirements of online learning. During this special period in China, self-study has become an important skill for medical students. Students of different majors have different learning styles. Dermatology students can review photographs of lesions to improve their skills in differential diagnosis. Internal medicine students can analyse complex cases to exercise their logical ability. Surgery students can learn more about internal medicine to become more comprehensive surgeons. Additionally, online learning allows students to restart long-forgotten projects, modify research papers and complete unfinished work. They can also review the literature in a field of interest, create an outline of future research and contemplate their career plan. All doctors in China are willing to apply for assistance from the National Natural Science Foundation of China, a famous and widely used research fund. Online application usually starts in March every year, but in 2020, it was postponed until April because of the epidemic. This gave medical students more time to carefully prepare for their application under the guidance of a mentor.

## Effective measures to ensure the health of medical students

Although the medical resources of the whole country are devoted to treatment of all patients infected with the novel coronavirus, the schools and government still make special efforts to protect the health of students. Peking Union Medical College has developed an online system called SARISenor, which is used by medical students to report the body temperature and physical condition every day. This system also has a locating function based on the global positioning system, which is convenient for localised management. Our medical school also developed a course to increase knowledge of COVID-19, and all students are required to study this course online. A test is administered after completion of the course, and students must complete the test to obtain a certificate and show the certificate to the school. This compulsory measure improves students’ awareness of the novel coronavirus and strengthens their ability to prevent COVID-19. With respect to psychological health, medical students are easily affected by disease-associated fear and pressure, and schools should be prepared to provide psychological services to those who need them.^6^ Students can also consult psychologists from university-affiliated hospitals who are online 24 hours a day. The Chinese government provides students with a wide coverage of virus protection education that has shown good results to date; the government also provides corresponding psychological counselling services. Specifically, China has^1^ stopped centralised classroom teaching,^2^ carried out antiepidemic knowledge training,^3^ encouraged the wearing of masks and^4^ paid attention to hand hygiene. These measures are worthy of implementation in foreign countries as well. Conversely, European countries have encouraged medical students to graduate early so that they may work to help fight COVID-19, which is worthy of implementation in China.

We cannot neglect the adverse effects of COVID-19 on Chinese scientific research. Fundamental experiments, scientific conferences, funding applications and other activities have been postponed or suspended because of the pandemic situation, which has caused a huge loss in scientific research in China. Specifically, pharmaceutical companies are lacking essential drugs because of shutdowns; scientific researchers are out of work because of the closures of laboratories; and students are unable to attain their academic degrees because of the suspension of research. However, the damage to science is insignificant compared with the level of human suffering. Notably, 5G wireless communication technology, artificial intelligence and cloud computing have played effective roles in prevention and monitoring during this epidemic emergency. Additionally, because of the lack of specific drugs and vaccines, traditional Chinese medicine has been adopted as a part of clinical therapy.

Thanks to the leadership of the government and the efforts of many medical workers, the effect of COVID-19 control in China has been remarkable. The Chinese Ministry of Education recently announced that senior medical students can return to universities in advance if circumstances permit. Doctors and postgraduate medical students are also glad to return to their clinical work and make their own contributions to the health of the people. With increased knowledge of the viral features, epidemiological characteristics, clinical symptoms and antivirus theory, efficient strategies have been taken to prevent, control and stop the spread of COVID-19. During the current COVID-19 pandemic, which is a worldwide war, everyone is a fighter. Under the close unity of all countries worldwide and with active participation of the world population, we believe that the prevention and control of COVID-19 will be finally achieved.
